# A regulatory variant in *TBX2 *promoter is related to the decreased susceptibility of congenital heart disease in the Han Chinese population

**DOI:** 10.1002/mgg3.530

**Published:** 2018-12-07

**Authors:** Ran‐ran Zhang, Ke Cai, Lian Liu, Qian Yang, Ping Zhang, Yong‐hao Gui, Feng Wang

**Affiliations:** ^1^ Department of Cardiology Children’s Hospital of Fudan University Shanghai China; ^2^ Department of Pediatrics the Affiliated Hospital of Qingdao University Qingdao China

**Keywords:** congenital heart disease, promoter, single‐nucleotide polymorphism, *TBX2*

## Abstract

**Background:**

Tbx2 plays a vital role in the cardiac cushion development. In this study, we aimed to determine the relationship between common genetic variants in the promoter region of *TBX2* gene and the risk of congenital heart disease (CHD).

**Methods:**

Blood samples of 516 CHD patients and 587 control subjects were enrolled. Sanger sequencing and SNaPshot analysis were performed for genotyping in our case–control cohort. Luciferase and electrophoretic mobility shift assay (EMSA) were conducted to uncover the potential modulatory mechanism of the related variants.

**Results:**

Variant rs4455026(c.‐1028G>C) in *TBX2* promoter region was found to be associated with significantly lower CHD susceptibility. The risk of CHD in C allele carriers (GC and CC genotypes) decreased by 30% compared to the wild‐type GG genotype subjects (OR = 0.70, 95% CI = 0.55–0.89, *p* = 0.0038). It was revealed that G to C variation resulted in a decrease in the transcriptional activity of luciferase gene, and a potential change in binding affinity with certain nucleoproteins in EMSA data.

**Conclusion:**

The minor C allele of rs4455026 in *TBX2* promoter region was related with lower CHD susceptibility in the Han Chinese population via repressing its transcriptional activity.

## 1 INTRODUCTION

Congenital heart disease (CHD) is the most common structural birth defect in human beings, affecting nearly 0.8% of live newborns worldwide (Edwards & Gelb, 2016). The etiology of CHD was not completely understood. Both inherited factors and environmental changes have been reported to induce CHD, among which a growing number of coding‐region mutations of certain genes, transcription factors in particular, were identified in CHD patients (Barua & Junaid, 2015; Fahed, Gelb, Seidman, & Seidman, 2013; Nuhrenberg, Gilsbach, Preissl, Schnick, & Hein, 2014; Schiano et al., 2015; Zaidi & Brueckner, 2017). Consequently, alterations in conformation or dosage of these genes could disturb transcriptional regulatory programs during cardiogenesis and resulted in heart malformations (Dorn & Matkovich, 2015; Meganathan, Sotiriadou, Natarajan, Hescheler, & Sachinidis, 2015).

Tbx2, a member of T‐box transcription factor family, is known to be essential for endocardial cushion formation. It is originally expressed in the cardiac crescent and then specifically restricted to the atrioventricular canal (AVC) and the outflow tract (OFT) region after the heart matured (Dupays, Kotecha, Angst, & Mohun, 2009; Harrelson et al., 2004). As a potential transcription repressor, Tbx2 also regulates the chamber‐specific gene expression, such as *Cx40*(*Gja5*), *Cx43*(*Gja1*), *Nppa,* and *Chisel*(*Smpx*) (Aanhaanen et al., 2011; Christoffels et al., 2004). Additionally, as a downstream target of Bmp2 signaling pathway, *Tbx2* contributes to the epithelial–mesenchymal transition process and the heart valve formation together with *Tgfβ2* (Shirai, Imanaka‐Yoshida, Schneider, Schwartz, & Morisaki, 2009). Spatiotemporal expression of *Tbx2* is strictly modulated throughout cardiogenesis and proved to be dosage‐dependent. *Tbx2‐*null mice embryos exhibited OFT defects and small AVC, whereas *Tbx2* transgenic mice generated relatively low cell proliferation in OFT and AVC regions (Harrelson et al., 2004). Also, mice fetus with aberrantly upregulated *Tbx2 *expression showed large linear heart tubes and failed to form chambers (Christoffels et al., 2004).

Dose changes of certain genes due to variants in their regulatory regions have been reported to be associated with CHD risk in human studies (Li et al., 2014, 2015). Our previous findings also demonstrated that single‐nucleotide polymorphisms (SNPs) in *TBX5*(601620) 3′UTR and *TBX20*(606061) promoter were related to increased and reduced CHD susceptibility by altering gene expression levels, respectively (Wang et al., 2017; Yu et al., 2016). Since few coding‐region mutations of *TBX2*(600747) were clarified in human samples, we speculated genetic factors in its regulatory region played a more significant role in pathogenesis of CHD. Both fragmental duplications and microdeletions containing *TBX2* were found in syndromic CHD cases (Ballif et al., 2010; Radio et al., 2010). Four novel rare variants in *TBX2* promoter region were identified in patients diagnosed with ventricular septal defects (Pang et al., 2013). However, it is unclear whether the common SNPs in *TBX2* promoter contribute to CHD susceptibility.

In the present study, we focused on the relationship between regulatory SNPs in *TBX2* promoter region and CHD susceptibility in a cohort comprising 516 CHD cases and 587 healthy control subjects in the Han Chinese population, uncovered the significantly associated variant and revealed the potential contributory mechanism in functional experiments.

## 2 MATERIALS AND METHODS

### 2.1 Ethical compliance

The study protocol was reviewed and approved by the ethics committee of Children’s Hospital of Fudan University. Written consents were obtained from the guardians of the children before study commencement.

### 2.2 Study subjects

All subjects were genetically ethnic Han Chinese. The CHD patients (*n* = 516, 1.59 ± 0.21 years), diagnosed by echocardiography or cardiac operation, were recruited from Children’s Hospital of Fudan University (Shanghai, China) between August 2015 and August 2016. Among them, cases of bicuspid aortic valve, patent foramen ovale, isolated patent ductus arteriosus, small‐size septal defects, and vascular malformations were excluded from the present study. Patients with syndromic disorders, systemic diseases, or familial CHD were not recruited. All the controls (*n* = 482, 5.41 ± 0.28 years) were non‐CHD children hospitalized for traumas in the Department of Orthopedics, Children’s Hospital of Fudan University. We also included 105 CHS (China South) samples from the 1,000 Genomes Project (http://browser.1000genomes.org/index.html) as controls, and the association study was ultimately based on 516 cases and 587 controls.

The CHD cases were classified into seven categories according to the commonly accepted criteria (Botto, Lin, Riehle‐Colarusso, Malik, & Correa, 2007). Specifically, 331 (64.1%) had septal defects, 39 (7.6%) had conotruncal defects, 19 (3.7%) had left ventricular outflow tract obstruction (LVOTO), 50 (9.7%) had right ventricular outflow tract obstruction (RVOTO), 12 (2.3%) had anomalous pulmonary venous return (APVR), 8 (1.6%) had complex CHD, and 57 (11.0%) had other cardiac abnormalities.

### 2.3 SNPs identification and genotyping

Twenty‐four CHD subjects and the equal number of controls were selected randomly to screen the SNPs in the promoter region of *TBX2* via sanger sequencing. The left 492 cases and 458 controls were genotyped by SNaPshot for SNPs with minor allele frequency (MAF) >5% and analyzed by Peak Scanner Software v1.0.

DNA was extracted from peripheral venous blood samples using a genomic DNA extraction kit (QIAGEN, China) and quantified by using NanoDrop 2000 (Thermo Fisher Scientific, USA). A fragment in the promoter region, covering approximately 1 kb upstream of *TBX2* (NG_052563.1) TSS (transcriptional start site), was amplified by PCR (Applied Biosystems 9700 PCR System, USA) and sequenced using Mutation Surveyor V4.0.8 (Applied Biosystems) in all samples. The PCR and sequencing primers are listed in Supporting Information Table S1.

### 2.4 Plasmid constructs, cell culture, and luciferase assays

To construct the reporter plasmids with *TBX2* promoter, we amplified a 992‐bp fragment containing either major G or minor C allele from human genomic DNA, and subcloned them into KpnI and XhoI restriction sites upstream of luciferase gene in pGL3‐basic vector (Promega, Madison, WI, USA). The recombinant plasmids were marked as pGL3‐G or pGL3‐C and verified by DNA sequencing. Primers are listed in Supporting Information Table S1.

Human embryonic kidney 293T (HEK 293T) cells, rat cardiac myocyte (H9c2) cells, and monkey kidney fibroblast‐like (COS‐7) cells were grown in Dulbecco’s Modified Eagle’s Medium (Invitrogen, USA) supplemented with 10% fetal bovine serum. HEK 293T (5.0 × 10^4^/ml), H9c2 (1.0 × 10^4^/ml), and COS‐7 (2.5 × 10^4^/ml) were seeded in 24‐well culture plates 24 hr before cell transfections. Transfections with 800 ng of each *TBX2* reporter plasmid (pGL3‐basic, pGL3‐G, and pGL3‐C) were conducted using Lipofectamine 3000 (Invitrogen) for each cell line. Luciferase assays were performed 24 hr later by using the Dual Luciferase Reporter Assay System (Promega) according to the manufacturer’s instructions.

### 2.5 Probe design and electrophoretic mobility shift assay

To predict the effect of genetic variants in *TBX2* promoter, two online bioinformatic algorithms were employed, including Alibaba (http://gene-regulation.com/pub/programs/alibaba2/index.html) and ALGGEN (https://alggen.lsi.upc.es/). Both tools demonstrated an alteration of nuclear protein binding at the locus of rs4455026. Thus, electrophoretic mobility shift assay (EMSA) probes labeled by FAM involving both of rs4455026 alleles were designed, namely, the Major‐probe with G allele and the Minor‐probe with C allele.

Nuclear extracts were isolated from HEK 293T and H9c2 cells using a nucleoprotein extraction kit (Beyotime, China). Protein concentration was measured using Enspire Multilabel Reader (PerkinElmer, USA) and stored at −70°C. The nuclear proteins were incubated with the probes and ran on a 6% polyacrylamide gel using the Typhoon FLA 9500 IP Laser Scanning Imager (GE healthcare, USA) to get the results.

### 2.6 Statistical analyses

The distribution of SNPs in control group needed to be in line with the Hardy–Weinberg equilibrium (HWE), that was, the allele (A/a) gene frequency (*p*, *q*): (*p* + *q*)^2^ = *p* ^2^ + 2*pq* + *q* ^2^ = 1. Therefore, HWE test in the controls and genotypic frequency disparity between the two groups was conducted using chi‐squared test. Odds ratios (ORs) and 95% confidence intervals (CIs) were calculated by unconditional logistic regression analyses and applied to evaluate associations between genotypes and CHD risk. Haplotype analysis among different SNPs loci was performed using Haploview 4.2 and SHEsis online analysis (http://analysis.bio-x.cn/myAnalysis.php).

Luciferase data were presented as mean ± standard deviation (*SD*). Independent *t* test was used to compare luciferase activities between the two groups using SPSS 19.0 software (SPSS, Chicago, IL, USA). The two‐tailed *p* < 0.05 was defined as statistical significance.

## 3 RESULTS

### 3.1 *TBX2* promoter variant rs4455026 significantly reduced CHD susceptibility in the Han Chinese population

In the present study, four variants were identified in the 1 kb of *TBX2 *promoter region. Three of them had MAF over 5% and thus were chosen for further SNaPshot genotyping, including rs1476781(c.‐1123T>C), rs4455026(c.‐1028G>C), and rs2286524(c.‐646C>T). In a total of 516 cases and 587 controls, variant rs4455026 was significantly correlated with reduced CHD susceptibility, with the C allele as the protective factor (*p* = 0.019; Table 1). Among the three SNPs in *TBX2* promoter, rs4455026 and rs2286524 were in strong linkage disequilibrium (*D*′ = 99% and *R* ^2^ = 88%), constituting three haplotypes with frequency more than 5% (Supporting Information Figure S1). However, there was no obvious association between the haplotypes and risk of CHD (Supporting Information Table S2). Therefore, rs4455026 was selected for further function study.

**Table 1:** Association of SNPs in *TBX2* promoter region with CHD risk in the case–control study

SNPs^a^	Genotype/Allele	Control	CHD	OR (95% CI)	*p *value	HWE‐*p*
rs1476781 (c.‐1123T>C)	CC	303	245	1	0.39	0.76
	CT	235	225	1.18 (0.92–1.52)		
	TT	49	46	1.16 (0.75–1.80)		
	CT–TT	284	271	1.18 (0.93–1.50)	0.17	
	C	841	715	1		
	T	333	317	1.12 (0.93–1.35)	0.23	
**rs4455026** a (c.‐1028G>C)	GG	296	305	1	**0.012**	1
	GC	242	170	**0.68 (0.53–0.89)**		
	CC	49	41	0.81 (0.52–1.27)		
	GC–CC	291	211	**0.70 (0.55–0.89)**	**0.0038**	
	G	834	780	1		
	C	340	252	**0.80 (0.66–0.96)**	**0.019**	
rs2286524 (c.‐646C>T)	CC	291	268	1	0.72	0.92
	CT	244	206	0.92 (0.71–1.18)		
	TT	52	42	0.88 (0.57–1.36)		
	CT–TT	296	248	0.91 (0.72–1.15)	0.43	
	C	826	742	1		
	T	348	29	0.93 (0.77–1.12)	0.43	

Note

CHD: congenital heart disease; CI: confidence interval; HWE: Hardy–Weinberg equilibrium; OR: odds ratio; SNPs: single‐nucleotide polymorphisms.

In bold, *p* < 0.05.

a SNPs in DNA sequence of *TBX2* promoter region (NG_052563.1).

To enhance the statistical power, we combined the rare homozygous CC with heterozygous GC genotype to compare with the wild‐type GG genotype in the dominant model of inheritance. According to the logistic regression analyses, GC and CC carriers had a significantly lower risk of CHD compared with the GG genotype subjects (OR = 0.70, 95% CI = 0.55–0.89, *p* = 0.0038). A similar result was indicated from the allele analysis that subjects with the minor C allele exhibited less distribution proportion in CHD cases than in controls (OR = 0.80, 95% CI = 0.66–0.96, *p* = 0.019).

### 3.2 *TBX2* c.‐1028G>C contributed to decreased risk of septal defects and outflow defects

To figure out the differential effect of variant rs4455026 on specific CHD types, we performed stratified analyses based on the previous CHD classifications. A statistical difference was observed in CHD subtypes of RVOTO (GC–CC vs. GG, OR = 0.396, 95% CI = 0.209–0.749, *p* = 0.003), as well as septal defects (GC–CC vs. GG, OR = 0.736, 95% CI = 0.561–0.966, *p* = 0.027; Table 2).

**Table 2:** Stratified analyses of rs4455026 by CHD classification

CHD classification	No.(GG vs. GC–CC)	*p *value	OR (95% CI)
Septal defects	192 versus 139	**0.027**	**0.736 (0.561–0.966)**
ASD	53 versus 27	**0.008**	**0.518(0.317–0.847)**
VSD	104 versus 94	0.609	0.919(0.666–1.269)
ASD + VSD	35 versus 18	**0.029**	**0.523(0.290–0.945)**
Conotruncal defects	24 versus 15	0.179	0.636 (0.327–1.236)
LVOTO	8 versus 11	0.475	1.399 (0.555–3.527)
RVOTO	36 versus 14	**0.003**	**0.396 (0.209–0.749)**
APVR	9 versus 3	0.092	0.339 (0.091–1.265)
Complex CHD	4 versus 4	0.981	1.017 (0.252–4.106)
Other cardiac abnormalities	32 versus 25	0.410	0.795 (0.460–1.374)

Notes

APVR: anomalous pulmonary venous return; CHD: congenital heart disease; CI: confidence interval; LVOTO: left ventricle outflow tract obstruction; OR: odds ratio; RVOTO: right ventricle outflow tract obstruction.

In bold, *p* < 0.05.

### 3.3 Variant c.‐1028G>C decreased *TBX2* promoter activity

**Figure 1:**
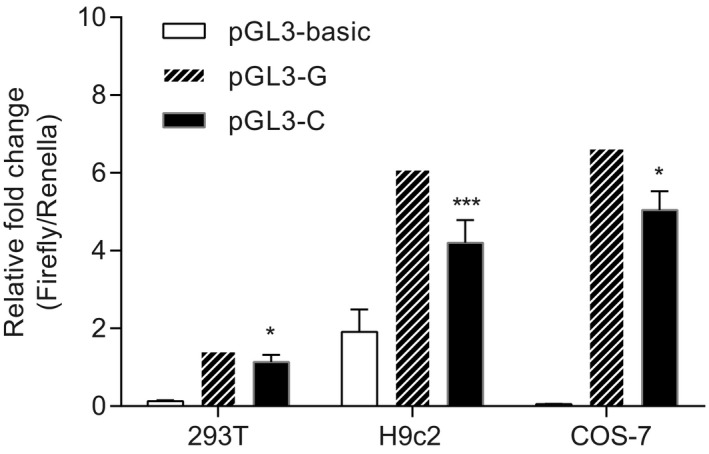
Luciferase assays to analyze transcriptional activity. Promoter activity analysis using dual‐reporter luciferase system in three cell lines. The columns represented the promoter activity of different groups. (*p < 0.05, ***p < 0.001)

Given the contribution of genetic variants in the promoter region to transcriptional activity, we constructed the recombined pGL3‐basic plasmids containing rs4455026 G (pGL3‐G) or C (pGL3‐C) allele to investigate promoter activity using luciferase assays. Compared to pGL3‐G transfection, transcriptional activity was decreased in pGL3‐C by 19.5%, 31%, and 15.1% for HEK 293T, H9c2, and COS‐7 cells, respectively (Figure 1). In combination with reduced CHD risk for the minor C allele, its repressed luciferase activity indicated that less expression level of *TBX2* might prevent CHD pathogenesis to some extent.

### 3.4 Minor C allele of rs4455026 repressed binding affinity of specific nuclear proteins

To further explore the mechanism of rs4455026 in interfering *TBX2* promoter activity, we predicted transcription factors changes in binding to this locus due to different alleles. It was suggested in Alibaba and ALGGEN that transcription enhancers Krox‐20 and Sp1 could bind with rs4455026 G allele, while G to C alteration diminished both factors’ binding affinity and instead, increased the binding affinity of a transcription inhibitor named "represso" (Supporting Information Figure S2).

**Figure 2:**
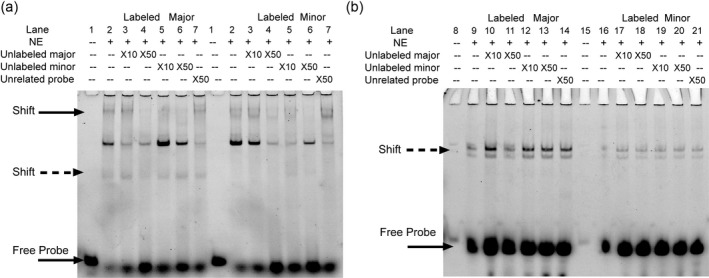
Analysis of DNA binding affinity with two alleles by EMSA in HEK 293T (a) and H9c2 (b) nuclear proteins. The solid and dotted arrows highlighted specific nuclear proteins that might interact with Major/Minor‐probes. Black and wide arrow pointed to the free probes

To verify our hypothesis, we designed FAM‐labeled probes Major (G allele) and Minor (C allele) to perform EMSA experiment using HEK‐293T and H9c2 cell extracts. Three bands were obtained in HEK 293T nuclear extracts. Among them, the band with solid arrows showed stronger binding of the Minor‐probe than the Major, whereas the band with dotted arrow presented weaker binding with the Minor‐probe (Figure 2a). In line with the findings in HEK 293T, competition assays in H9c2 also indicated lower binding affinity of nuclear transcription factors to the minor allele (Figure 2b), and the bindings of the labeled probes to the extracts were inhibited by higher concentration of unlabeled probes in both cell lines. According to the EMSA result, we presumed that transcription stimulators might overweigh the repressors in nuclear proteins to bind the Minor‐C probe, thus resulting in lower *TBX2* promoter activity for C allele carriers in CHD cases.

## 4 DISCUSSION

As one of the key genes participating in the formation of endocardial cushions, few coding‐region mutations of *TBX2* have been reported in sporadic CHD. In contrast, four novel rare mutations in *TBX2* promoter region were indicated to cause ventricular septal defects, indicating the contribution of *TBX2* regulatory variants to the occurrence of CHD (Pang et al., 2013). In accordance with the previous studies, both duplications and microdeletions of the chromosome fragments containing *TBX2* (located at chromosome 17q23) could result in syndromic disorders including heart defects (Ballif et al., 2010; Radio et al., 2010). Taken together with the aberrant cardiogenesis in mice models, the function of Tbx2 is dosage sensitive throughout the cardiac embryogenesis (Aanhaanen et al., 2011; Harrelson et al., 2004). Therefore, we speculated that common regulatory variants of *TBX2* might play a role in CHD risk in the condition of altering *TBX2* expression levels. In our cohort composed of 516 CHD children and 587 control subjects in the Han Chinese population, association studies were conducted to investigate the association between common SNPs in *TBX2* promoter and the susceptibility of CHD. A significantly lower CHD risk was revealed for rs4455026 C carriers than wild‐type GG subjects (OR = 0.70, 95% CI = 0.55–0.89, *p* = 0.0038), notably in RVOTO (OR = 0.396, 95% CI = 0.209–0.749, *p* = 0.003) and septal defects (OR = 0.736, 95% CI = 0.561–0.966, *p* = 0.027). This result was consistent with the expression pattern of *Tbx2*, mostly restricted in the AVC and OFT region, which was indispensable for cardiac cyclization and the right ventricle formation (Aanhaanen et al., 2009).

Variants in promoter region were reported to influence genes’ transcriptional activity (Yu et al., 2016). In our study, functional analyses using luciferase assays and EMSA indicated the rs4455026 C allele might decrease *TBX2* promoter activity by altering the binding affinity of certain transcription factors. According to the online bioinformatic tools, Krox‐20 and Sp1 were predicted as potential stimulators with lower binding affinity with C allele, whereas another inhibitory factor called “represso” had higher binding affinity (Desmazieres, Charnay, & Gilardi‐Hebenstreit, 2009; Martin‐Gallausiaux et al., 2018). We inferred that the stimulators had more significant influence than the inhibitor, and as a result, G to C alteration gave rise to a lower *TBX2* promoter activity and less gene expression.

Experimental evidence based on animal models has confirmed that *Tbx2 *modulates the endocardial cushion development in a dose‐dependent manner, for both deletion and upregulation of *Tbx2* interfere AVC formation (Chi et al., 2008; Singh et al., 2009). In particular, *Tbx2* transgenic mice failed to form chambers and generated low cell proliferation in OFT and AVC regions (Harrelson et al., 2004). In our study, rs4455026 C allele showed a protective effect to decrease CHD risk via reducing gene’s transcriptional activity, which implied overexpression of *TBX2* could also disturb heart development. A patient with two copies of *TBX2*, caused by the chromosome 17q23.2 duplication, was reported to suffer from complex congenital heart defects (Liu et al., 2018). The underlying mechanism might be that loss of repressive modulation of *Tbx2* on *Nkx2.5*, which further targets the chamber‐specific genes involving *Nppa* and *Cx43*, results in abnormal formation of cardiac chambers and septation (Christoffels et al., 2004). As a transcription repressor, *Tbx2* can also decrease the proliferation of non‐chamber myocardium by inhibiting *N*‐myc1 and avoids the deposition of cardiac glia in the ventricles by decreasing the expressions of *Has2* and *Tgfβ2*, which are important for normal shaping of heart structures (Shirai et al., 2009). Besides, Tbx2 acts to inhibit Tbx5‐induced chamber differentiation and endocardial cushion formation, making these regions to develop another fate (Habets et al., 2002). These data contribute to interpret the reason why lower *TBX2* expression decreased the susceptibility of CHD in the present study.

Several limitations were observed in our study. First, since patient amounts of several CHD subtypes were relatively scarce, such as LVOTO and APVR, more samples are needed to optimize the statistical power for stratified analyses. Furthermore, the lack of heart tissues and specific antibodies also limited the mechanism studies concerning rs4455026 modulation on *TBX2* promoter activity. Future investigations are deserved to clarify the specific regulatory nuclear proteins and to provide a new clue to reveal the etiology of CHD.

To conclude, the minor C allele of rs4455026 in *TBX2* promoter region was associated with reduced CHD susceptibility in the Han Chinese population, especially for the CHD subtypes of RVOTO and septal defects. Functional analyses in three cell lines demonstrated that G to C variation resulted in decreased transcriptional activity and lower binding ability with some nuclear proteins. Our study illustrated a potential role of SNPs in the regulatory region of *TBX2* in affecting its transcriptional activity and revealed a possible mechanism for the pathogenesis of sporadic CHD.

## CONFLICT OF INTEREST

None declared.

## References

[mgg3530-bib-0001] Aanhaanen, W. T. , Boukens, B. J. , Sizarov, A. , Wakker, V. , de Gier‐de Vries, C. , van Ginneken, A. C. , … Christoffels, V. M. (2011). Defective Tbx2‐dependent patterning of the atrioventricular canal myocardium causes accessory pathway formation in mice. Journal of Clinical Investigation, 121(2), 534–544. 10.1172/JCI44350 21266775 PMC3026729

[mgg3530-bib-0002] Aanhaanen, W. T. , Brons, J. F. , Dominguez, J. N. , Rana, M. S. , Norden, J. , Airik, R. , … Christoffels, V. M. (2009). The Tbx2+ primary myocardium of the atrioventricular canal forms the atrioventricular node and the base of the left ventricle. Circulation Research, 104(11), 1267–1274. 10.1161/circresaha.108.192450 19423846

[mgg3530-bib-0003] Ballif, B. C. , Theisen, A. , Rosenfeld, J. A. , Traylor, R. N. , Gastier‐Foster, J. , Thrush, D. L. , … Shaffer, L. G. (2010). Identification of a recurrent microdeletion at 17q23.1q23.2 flanked by segmental duplications associated with heart defects and limb abnormalities. American Journal of Human Genetics, 86(3), 454–461. 10.1016/j.ajhg.2010.01.038 20206336 PMC2833380

[mgg3530-bib-0004] Barua, S. , & Junaid, M. A. (2015). Lifestyle, pregnancy and epigenetic effects. Epigenomics, 7(1), 85–102. 10.2217/epi.14.71. 25687469

[mgg3530-bib-0005] Botto, L. D. , Lin, A. E. , Riehle‐Colarusso, T. , Malik, S. , & Correa, A. (2007). Seeking causes: Classifying and evaluating congenital heart defects in etiologic studies. Birth Defects Research Part A: Clinical and Molecular Teratology, 79(10), 714–727. 10.1002/bdra.20403 17729292

[mgg3530-bib-0006] Chi, N. C. , Shaw, R. M. , De Val, S. , Kang, G. , Jan, L. Y. , Black, B. L. , & Stainier, D. Y. (2008). Foxn4 directly regulates tbx2b expression and atrioventricular canal formation. Genes & Development, 22(6), 734–739. 10.1101/gad.1629408 18347092 PMC2275426

[mgg3530-bib-0007] Christoffels, V. M. , Hoogaars, W. M. , Tessari, A. , Clout, D. E. , Moorman, A. F. , & Campione, M. (2004). T‐box transcription factor Tbx2 represses differentiation and formation of the cardiac chambers. Developmental Dynamics, 229(4), 763–770. 10.1002/dvdy.10487 15042700

[mgg3530-bib-0008] Desmazieres, A. , Charnay, P. , & Gilardi‐Hebenstreit, P. (2009). Krox20 controls the transcription of its various targets in the developing hindbrain according to multiple modes. Journal of Biological Chemistry, 284(16), 10831–10840. 10.1074/jbc.M808683200 19218566 PMC2667770

[mgg3530-bib-0009] Dorn II, G. W. , & Matkovich, S. J. (2015). Epitranscriptional regulation of cardiovascular development and disease. Journal of Physiology, 593(8), 1799–1808. 10.1113/jphysiol.2014.283234 25433070 PMC4405743

[mgg3530-bib-0010] Dupays, L. , Kotecha, S. , Angst, B. , & Mohun, T. J. (2009). Tbx2 misexpression impairs deployment of second heart field derived progenitor cells to the arterial pole of the embryonic heart. Developmental Biology, 333(1), 121–131. 10.1016/j.ydbio.2009.06.025 19563797

[mgg3530-bib-0011] Edwards, J. J. , & Gelb, B. D. (2016). Genetics of congenital heart disease. Current Opinion in Cardiology, 31(3), 235–241. 10.1097/HCO.0000000000000274 26872209 PMC4868504

[mgg3530-bib-0012] Fahed, A. C. , Gelb, B. D. , Seidman, J. G. , & Seidman, C. E. (2013). Genetics of congenital heart disease: The glass half empty. Circulation Research, 112(4), 707–720. 10.1161/circresaha.112.300853 23410880 PMC3827691

[mgg3530-bib-0013] Habets, P. E. , Moorman, A. F. , Clout, D. E. , van Roon, M. A. , Lingbeek, M. , van Lohuizen, M. , … Christoffels, V. M. (2002). Cooperative action of Tbx2 and Nkx2.5 inhibits ANF expression in the atrioventricular canal: Implications for cardiac chamber formation. Genes & Development, 16(10), 1234–1246. 10.1101/gad.222902 12023302 PMC186286

[mgg3530-bib-0014] Harrelson, Z. , Kelly, R. G. , Goldin, S. N. , Gibson‐Brown, J. J. , Bollag, R. J. , Silver, L. M. , & Papaioannou, V. E. (2004). Tbx2 is essential for patterning the atrioventricular canal and for morphogenesis of the outflow tract during heart development. Development, 131(20), 5041–5052. 10.1242/dev.01378 15459098

[mgg3530-bib-0015] Li, C. , Li, X. , Pang, S. , Chen, W. , Qin, X. , Huang, W. , … Yan, B. (2014). Novel and functional DNA sequence variants within the GATA6 gene promoter in ventricular septal defects. International Journal of Molecular Sciences, 15(7), 12677–12687. 10.3390/ijms150712677 25036032 PMC4139867

[mgg3530-bib-0016] Li, P. , Li, H. , Zheng, Y. , Qiao, B. , Duan, W. , Huang, L. , … Wang, H. (2015). Variants in the regulatory region of WNT5A reduced risk of cardiac conotruncal malformations in the Chinese population. Scientific Reports, 5, 13120 10.1038/srep13120 26278011 PMC4538571

[mgg3530-bib-0017] Liu, N. , Schoch, K. , Luo, X. , Pena, L. D. M. , Bhavana, V. H. , Kukolich, M. K. , … Yamamoto, S. (2018). Functional variants in TBX2 are associated with a syndromic cardiovascular and skeletal developmental disorder. Human Molecular Genetics, 27(14), 2454–2465. 10.1093/hmg/ddy146 29726930 PMC6030957

[mgg3530-bib-0018] Martin‐Gallausiaux, C. , Beguet‐Crespel, F. , Marinelli, L. , Jamet, A. , Ledue, F. , Blottiere, H. M. , & Lapaque, N. (2018). Butyrate produced by gut commensal bacteria activates TGF‐beta1 expression through the transcription factor SP1 in human intestinal epithelial cells. Scientific Reports, 8(1), 9742 10.1038/s41598-018-28048-y 29950699 PMC6021401

[mgg3530-bib-0019] Meganathan, K. , Sotiriadou, I. , Natarajan, K. , Hescheler, J. , & Sachinidis, A. (2015). Signaling molecules, transcription growth factors and other regulators revealed from in‐vivo and in‐vitro models for the regulation of cardiac development. International Journal of Cardiology, 183, 117–128. 10.1016/j.ijcard.2015.01.049 25662074

[mgg3530-bib-0020] Nuhrenberg, T. , Gilsbach, R. , Preissl, S. , Schnick, T. , & Hein, L. (2014). Epigenetics in cardiac development, function, and disease. Cell and Tissue Research, 356(3), 585–600. 10.1007/s00441-014-1887-8 24817102

[mgg3530-bib-0021] Pang, S. , Liu, Y. , Zhao, Z. , Huang, W. , Chen, D. , & Yan, B. (2013). Novel and functional sequence variants within the TBX2 gene promoter in ventricular septal defects. Biochimie, 95(9), 1807–1809. 10.1016/j.biochi.2013.05.007 23727221

[mgg3530-bib-0022] Radio, F. C. , Bernardini, L. , Loddo, S. , Bottillo, I. , Novelli, A. , Mingarelli, R. , & Dallapiccola, B. (2010). TBX2 gene duplication associated with complex heart defect and skeletal malformations. American Journal of Medical Genetics Part A, 152A(8), 2061–2066. 10.1002/ajmg.a.33506 20635360

[mgg3530-bib-0023] Schiano, C. , Vietri, M. T. , Grimaldi, V. , Picascia, A. , De Pascale, M. R. , & Napoli, C. (2015). Epigenetic‐related therapeutic challenges in cardiovascular disease. Trends in Pharmacological Sciences, 36(4), 226–235. 10.1016/j.tips.2015.02.005 25758254

[mgg3530-bib-0024] Shirai, M. , Imanaka‐Yoshida, K. , Schneider, M. D. , Schwartz, R. J. , & Morisaki, T. (2009). T‐box 2, a mediator of Bmp‐Smad signaling, induced hyaluronan synthase 2 and Tgfbeta2 expression and endocardial cushion formation. Proceedings of the National Academy of Sciences of the United States of America, 106(44), 18604–18609. 10.1073/pnas.0900635106 19846762 PMC2773962

[mgg3530-bib-0025] Singh, R. , Horsthuis, T. , Farin, H. F. , Grieskamp, T. , Norden, J. , Petry, M. , … Kispert, A. (2009). Tbx20 interacts with smads to confine tbx2 expression to the atrioventricular canal. Circulation Research, 105(5), 442–452. 10.1161/circresaha.109.196063 19661464

[mgg3530-bib-0026] Wang, F. , Liu, D. , Zhang, R.‐R. , Yu, L.‐W. , Zhao, J.‐Y. , Yang, X.‐Y. , … Wang, H.‐Y. (2017). A TBX5 3'UTR variant increases the risk of congenital heart disease in the Han Chinese population. Cell Discovery, 3, 17026 10.1038/celldisc.2017.26 28761722 PMC5527299

[mgg3530-bib-0027] Yu, L. W. , Wang, F. , Yang, X. Y. , Sun, S. N. , Zheng, Y. F. , Li, B. B. , … Wang, H. Y. (2016). Mild decrease in TBX20 promoter activity is a potentially protective factor against congenital heart defects in the Han Chinese population. Scientific Reports, 6, 23662 10.1038/srep23662 27034249 PMC4817057

[mgg3530-bib-0028] Zaidi, S. , & Brueckner, M. (2017). Genetics and genomics of congenital heart disease. Circulation Research, 120(6), 923–940. 10.1161/circresaha.116.309140 28302740 PMC5557504

