# Supplementary material for: A regulatory variant in TBX2 promoter is related to the decreased susceptibility of congenital heart disease in the Han Chinese population
Source: Mol Genet Genomic Med. 2018 Dec 7;7(2):e00530. doi: 10.1002/mgg3.530 (PMC6393683; doi:10.1002/mgg3.530)
Supplement: Supplementary file 1 [file MGG3-7-na-s001.docx]

SUPPLEMENTARY MATERIAL

**Supplementary Table S1. DNA or RNA sequence of all used primers.**

| Primer Name | Sequence (5’ to 3’) | Assay |
| --- | --- | --- |
| *TBX2*-promoter-F | TGCGCTTCCGAGAGATGAG | PCR/Sequence |
| *TBX2*- promoter-R | TCGGAAGCCTCAGCAGTAAGA | PCR/Sequence |
| c.-1123T>C -JC | aaagagctttccagcggccgggaggagcgg | SNaPshot Genotying |
| c.-1028G>C -JC | aaaaaaaaaaaaagagtcttccggtgaaggcgggcgcgg | SNaPshot Genotying |
| c.-646C>T-JC | aaaaaaaaaaaaaaaaaaaaaaaaaaaaaaaaaaaaaaaaaaaaactgcgggtccttccccaaggccccgggacc | SNaPshot Genotying |
| *TBX2*-KpnI-F | CGGGGTACCCCG acgcgttactcagcactcct | Clone Construct |
| *TBX2*-Xhol-R | CCGCTCGAGCGG tttggaccaattgtgggtct | Clone Construct |
| c.-1123T>C F | GACCCGCTCCTCCCGGCCGCTGGAAAGC | Point Mutation |
| c.-1123T>C R | GGGTCCTCGGGGCCAGGAAGGTGAGCGC | Point Mutation |
| c.-1028G>C F | GGTGAAGGCGGGCGCGGCTAGGGGGTCCC | Point Mutation |
| c.-1028G>C R | GCCGCGCCCGCCTTCACCGGAAGACTCGGA | Point Mutation |
| c.-646C>T F | ACCCGGGCTCCCCTCGCCTCAGGCCCTTTC | Point Mutation |
| c.-646C>T R | CCGGGTCCCGGGGCCTTGGGGAAGGACC | Point Mutation |
| c.-1028 G-F | gtgaaggcgggcgcgggtagggggtccccgcgt | EMSA probe |
| c.-1028 G-R | acgcggggaccccctacccgcgcccgccttcac | EMSA probe |
| c.-1028 C-F | gtgaaggcgggcgcggCtagggggtccccgcgt | EMSA probe |
| c.-1028 C-R | acgcggggaccccctaGccgcgcccgccttcac | EMSA probe |

**Supplementary Table S2. The association analysis of the haplotypes in *TBX2* promoter region with the risk of CHD.**

| **rs1476781**  **（**c.-1123T>C**）** | | **rs4455026**  **(**c.-1028G>C**)** | **rs2286524**  (c.-646C>T) | **Freq** | **OR (95% CI)** | ***P*-value** |
| --- | --- | --- | --- | --- | --- | --- |
| 1 | C | G | C | 0.421 | 1.00 | --- |
| 2 | T | G | C | 0.288 | 1.06 (0.86 - 1.30) | 0.6 |
| 3 | C | C | T | 0.267 | 0.87(0.71 - 1.07) | 0.18 |
| rare | * | * | * | 0.025 | 3.11(1.59 - 6.08) | <0.0001 |
| Global haplotype association *P*-value: <0.0001 | | | | | | |

**Supplementary Figure S1. Linkage disequilibrium of three SNPs in *TBX2* promoter region in all samples.** Each box represented the LD relationship between neighboring SNPs, the left schematic diagram was D’ value and the right schematic diagram was r^2^ value. The white line on the top showed the relative physical positions of the SNPs on the chromosome.

**
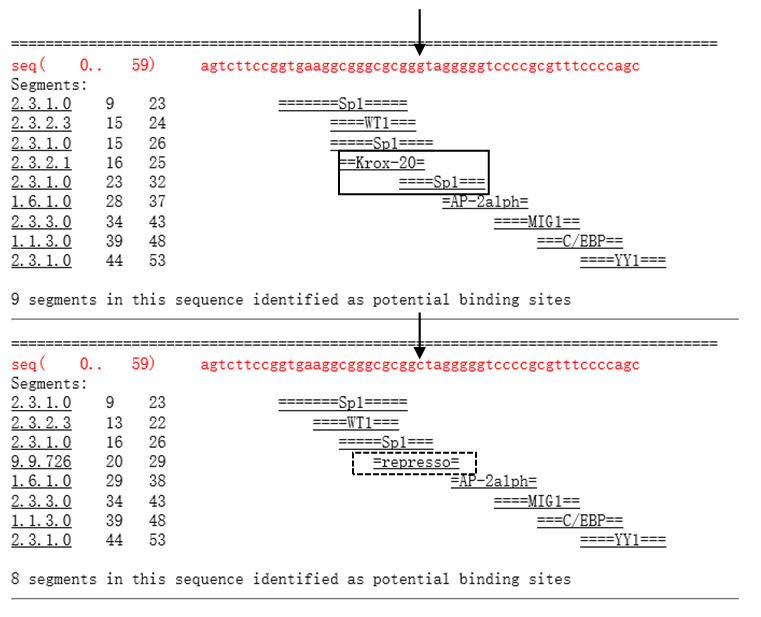
**

**Supplementary Figure S2. Predicted transcriptional factors binding with rs4455026 alleles.** Arrows point to rs4455026 site. Solid box represented specific TFs binding with G allele and dotted box represented specific TF binding with C allele.
